# Supplementary material for: Kinetic Modeling of Time-Dependent Enzyme Inhibition by Pre-Steady-State Analysis of Progress Curves: The Case Study of the Anti-Alzheimer’s Drug Galantamine
Source: Int J Mol Sci. 2022 May 3;23(9):5072. doi: 10.3390/ijms23095072 (PMC9105972; doi:10.3390/ijms23095072)
Supplement: Supplementary file 1 [file ijms-23-05072-s001.zip › Figura S1.pdf]

Supplementary Information

Kinetic Modeling of Time-Dependent Enzyme Inhibition by Pre-Steady-State Analysis of Progress Curves: The Case Study of the Anti-Alzheimer's Drug Galantamine

Doriano Lamba and Alessandro Pesaresi *

Istituto di Cristallografia – Consiglio Nazionale delle Ricerche, Trieste, Italy

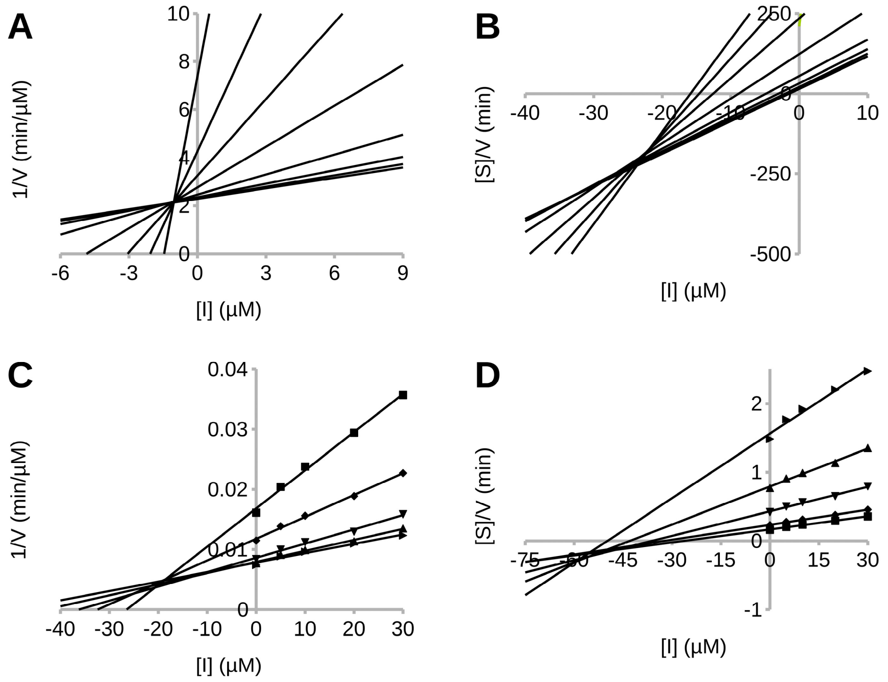

Figure S1. Deconvolution of competitive (*K_{ic}*) and uncompetitive (*K_{iu}*) component of mixed-type inhibition. Dixon plots of 1/V against different concentration of inhibitors [I] at various concentrations of substrate (**A**,**C**) and Cornish–Bowden plots of [Substrate]/V against inhibitor concentration [I] at various substrate concentrations (**B**,**D**). Analysis of synthetic data for enzyme inhibition by an inhibitor with k_{off} of $3\cdot10^{-2}$ s^{-1} (**A**,**B**). Analysis of substrate-initiated reactions of *Tc*AChE inhibited by galantamine (**C**,**D**). In Dixon plots lines intersect at x-coordinate equal to $-1/K_{ic}$ and in Cornish-Bowden plot at x-coordinate equal to $-1/K_{iu}$.
